# Functional levels and MRI patterns of muscle involvement in upper limbs in Duchenne muscular dystrophy

**DOI:** 10.1371/journal.pone.0199222

**Published:** 2018-06-20

**Authors:** Claudia Brogna, Lara Cristiano, Tommaso Tartaglione, Tommaso Verdolotti, Lavinia Fanelli, Luana Ficociello, Giorgio Tasca, Roberta Battini, Giorgia Coratti, Nicola Forcina, Roberto De Santis, Giulia Norcia, Sara Carnicella, Cesare Colosimo, Pierre Carlier, Marika Pane, Eugenio Mercuri

**Affiliations:** 1 Pediatric Neurology and Nemo Clinical Centre, Fondazione Policlinico Universitario "A. Gemelli IRCSS", Università Cattolica del sacro Cuore, Rome, Italy; 2 Department of Radiology, Fondazione Policlinico Universitario "A. Gemelli IRCSS", Università Cattolica del sacro Cuore, Rome, Italy; 3 Institute of Neurology, Fondazione Policlinico Universitario "A. Gemelli IRCSS", Università Cattolica del sacro Cuore, Rome, Italy; 4 IRCSS Stella Maris, Pisa, Italy; 5 AIM and CEA NMR laboratory, Institute of Myology, Paris, France; Universite de Nantes, FRANCE

## Abstract

The aim of the study was to evaluate the spectrum of upper limb functional activities and imaging finding in a cohort of patients affected by Duchenne muscular dystrophy. Thirty-one patients of age between 5 and 29 years were included in the study (17 ambulant and 14 non-ambulant). They were all assessed using the Performance of Upper Limb (PUL) test and muscle MRI of shoulder, arm and forearm in order to establish if the functional scores obtained at shoulder, mid and distal level related to specific patterns of involvement in each upper limb segment on muscle MRI. At shoulder level, latissimus dorsi, serratus anterior, infraspinatus and subscapularis were always involved, even in patients with full functional scores at shoulder level. Diffuse and severe involvement of all muscles was found in the patients with a PUL shoulder functional score of ≤ 5. At arm level biceps brachii, brachialis and triceps were generally concordantly involved or spared. Some degree of involvement could already be detected in patients with reduced scores on the PUL mid domain. They were generally severely involved in patients with functional scores less than 6 at mid-level. At distal level supinator and pronator muscles were often involved, followed by brachioradialis and, less frequently, by the muscles of the flexor compartment. The extensor muscles were generally completely spared. A diffuse and severe involvement was found only in patients who had very low scores (8 or below) on the PUL distal domain. The integrated use of functional scales and imaging allowed to establish patterns of involvement at each level, and the functional scores that were more frequently associated with diffuse and severe involvement.

## Introduction

Over the last few years, increasing attention has been devoted to the assessment of upper limbs in Duchenne muscular dystrophy (DMD) [1–13]. Several studies have used generic scales to assess upper limb function[11] but, more recently, disease specific tools have been proposed [2, 3, 14]. The Performance of Upper Limb (PUL) test has been specifically designed to assess the progression of involvement in both ambulant and non-ambulant DMD patients [1–3, 15].

So far, both cross sectional and longitudinal studies have shown that the PUL can reliably follow the proximal to distal progression typically observed in DMD [1–3, 15]. Early signs of proximal weakness and abnormal function at shoulder level muscle involvement can already be detected in ambulant boys with a predictable decline subsequently involving elbow and distal domains after loss of ambulation. No systematic study has been performed to establish whether the different functional profiles observed on the PUL are related to specific patterns of muscle involvement on MRI at all three levels.

So far, only few studies have explored upper limb involvement using muscle Magnetic Resonance Imaging (MRI) and in some cases, also MR spectroscopy [4, 8, 16, 17]. These studies have provided important qualitative and quantitative information but mainly focused on the assessment of one level, either distal or proximal.

The aim of the present study was to evaluate whether the functional involvement, detected on the PUL, is related to muscle involvement using a wider MRI protocol that includes shoulder, arm and forearm. This was assessed in a cohort of DMD patients ranging from young boys to young adults, therefore including both ambulant and non-ambulant patients. More specifically, we wished to establish if the PUL scores in each functional domain relate to specific patterns of involvement in each upper limb segment on MRI and if early signs of muscle involvement can be detected even in patients with full functional scores in the correspondent segment.

## Methods

The study is part of a project aimed at establishing functional and muscle MRI changes in DMD. The study was approved by the Ethics committee of the Fondazione Gemelli, Rome. Written informed consent was obtained for all the patients who agreed to participate. For the minors, consent was obtained by their parents. As part of this, we enrolled DMD patients attending their routine follow up clinics between September 2016 and August 2017. The exclusion criteria were related to the impossibility to perform MRI without sedation, therefore excluding very young children or those with severe cognitive or behavioral problems. We also excluded patients with severe joint contractures, pacemakers, respiratory or cardiac problems that would interfere with positioning or performing MRI.

### PUL 2.0

The PUL 2.0 (S1 File) includes an entry item to define the starting functional level, and 22 items subdivided into shoulder level (6 items), mid-level (9 items) and distal level (7 items) dimension [2]. The entry items are based on a revised version of the Brooke score and range from score 0 –no useful hand function—to score 6 full shoulder abduction–no weakness. For weaker patients a low score on the entry item means high level items do not need to be performed. Each dimension (shoulder, mid, distal) can be scored separately.

In the PUL 2.0 there is a maximum score of 12 for the shoulder level, 17 for the middle level, and 13 for the distal level. A total score can be achieved by adding the three level scores (max global score 42). Details of the training sessions and of the reliability studies have already been reported for the original PUL version [1].

### Muscle MRI

Unilateral upper-limb MRI was performed at 1.5T (Philips Ingenia) using a flexible body coil (body SENSE 32 Channel Flex Coil). Subjects lay in the scanner in the head-first supine position, with the dominant upper limb to be imaged lying in a comfortable position on the scanner bed alongside the torso. The upper limb was stabilized using a fabricated thermoplastic splint and sandbags were placed over the forearm and hand to minimize motion. Boys were encouraged to watch a movie or cartoon during scan acquisition and usually a parent and/or a staff member was present in the scan room during scan acquisition. The patients did not receive any sedation and the total examination time was approximately 20 to 30 minutes.

Non contrast-enhanced images were obtained from the dominant upper limb.

TSE T1 -weighted spin-echo was acquired on axial plane selected in respect to the long axis of the humerus for the shoulder and arm, and in respect of the long axis of the radius for the forearm. The slices were set up to cover the entire extension of the upper limb. Each muscle was evaluated throughout its length.

Scan parameters were as follows: TSE T1 (FOV 160x160mm, voxel 0.8x0.8, matrix 200x200, 5mm axial slices, slice gap 0mm, flip angle 90°, TR 150ms, TE 8ms).

Descriptive analysis was used to identify the muscles that were more frequently affected in the different segments. At shoulder level the muscles examined were: deltoid, supraspinatus, infraspinatus, subscapularis, coraco-brachialis, pectoralis major and minor, teres minor, latissimus dorsi, serratus anterior. At arm level the muscles examined were: biceps brachii, brachialis and triceps brachii. At the forearm level the muscles examined were: supinator, pronator teres, flexor carpi radialis, palmar, flexor digitorum superficialis, flexor carpi ulnaris, flexor digitorum profundus, anconeus, flexor pollicis longus, extensor carpi ulnaris, extensor digiti minimi, extensor digitorum, extensor carpi radialis, brachioradialis, extensor pollicis longus.

For each segment, we identified the muscles that were more often spared or affected. Although in the present paper we did not aim to quantify the level of involvement, as this will be separately reported, we used a previously reported classification to have a rough estimate of the level of involvement. All muscles MRI scans were assessed for normal or abnormal signal intensity within the different muscles groups and scored using Mercuri classification [18–20], as follows:

Stage 0: normal appearance; Stage 1: scattered small areas of increased intensity on T1W images;
Stage 2a: numerous discrete areas of increased intensity on T1W images involving less than 30% of the volume of the muscle; Stage 2b: numerous discrete areas of increased intensity on T1W images with early confluence of, 30–60% of the volume of the muscle; Stage 3: washed-out appearance due to confluent areas of increased intensity on T1W images with muscle still present at the periphery; Stage 4: end-stage appearance, muscle entirely replaced by areas of increased intensity on T1W images. We arbitrarily subdivided muscles with normal MRI or with only minimal changes (score 0 and 1), those with intermediate involvement (grades 2 to 3) and those with complete replacement (grade 4). Three examiners (LC, TT and TV) scored the scans separately with consensus on the scores of over 90%. In the discordant cases the scans were reviewed by all examiners and an agreement was found.

## Results

Thirty-one patients were included in the study. Their age ranged between 5 and 29 years (mean 12.7 SD: ±5.5). Seventeen were ambulant (age range 5–15) and 14 were non-ambulant (age range 10–29). All the patients were on steroids.

### PUL 2.0

**Fig 1:**
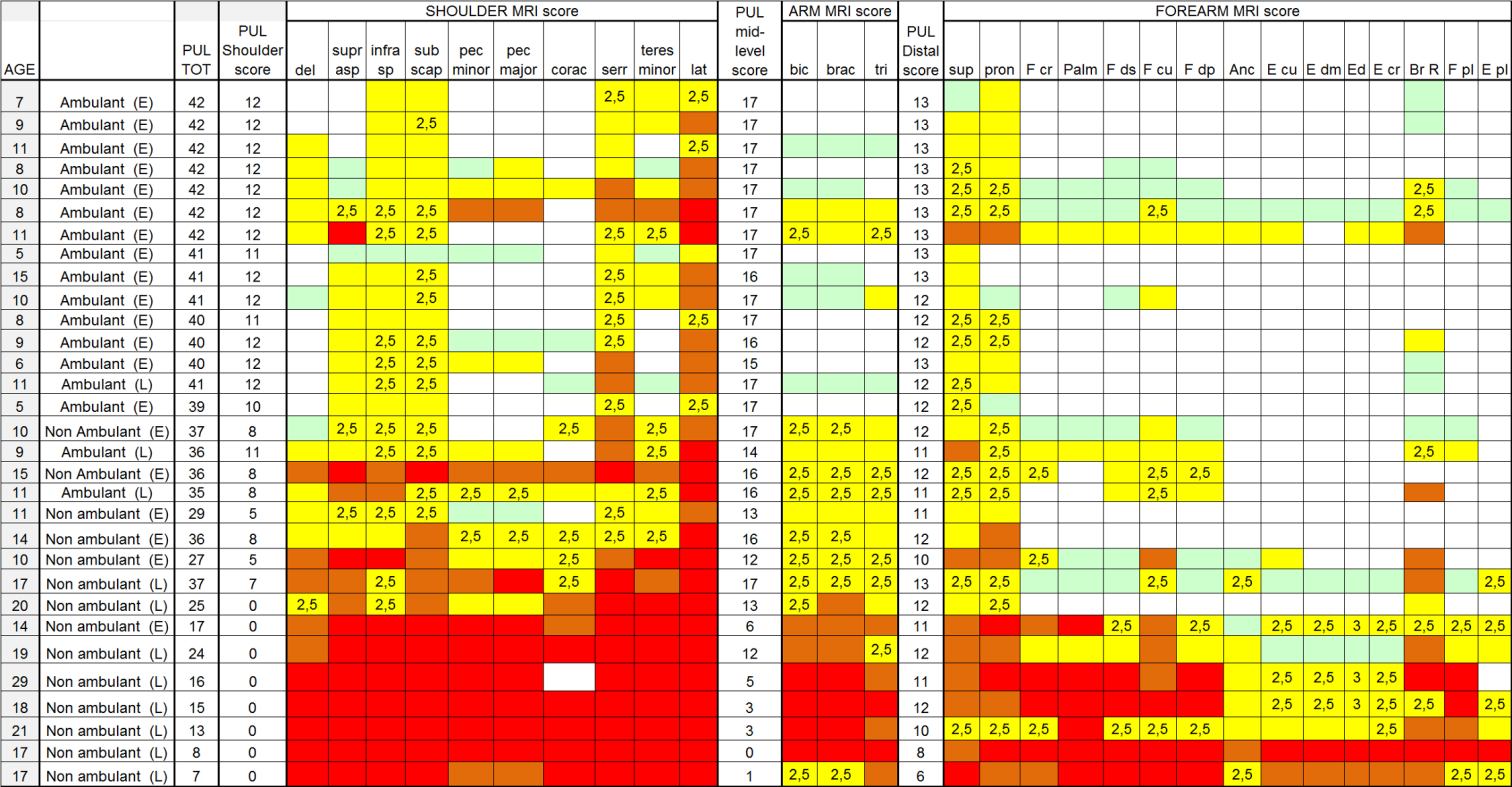
Individual details of imaging and PUL findings at shoulder, arm and forearm level. The shading reflects the severity of involvement with the score of 0 shown as a white cell, score of 1 as pale green, score of 2 as yellow, score of 3 as orange and score of 4 as red. The grade 2.5 was used to identify patients with 2b involvement. Del = deltoid; suprasp = supraspinatus; infrasp = infraspinatus; subscap = subscapularis; pec = pectoralis; corac = coraco-brachialis; serr = serratus anterior; lat = latissimus dorsi; bic = biceps brachii; brac = brachialis;tri = triceps brachii; sup = supinator;pron = pronator teres;F cp = flexor carpi radialis; palm = palmar; F ds = flexor digitorum superficialis; F cu = flexor carpi ulnaris; Fdp = flexor digitorum profundus; Anc = anconeus; E cu = extensor carpi ulnaris; Edm = extensor digiti minimi; E d = extensor digitorum; E cr = extensor carpi radialis; Br R = brachioradialis; F pl = flexor pollicis longus; E pl = extensor pollicis longus. Ambulant (E) = ambulant early; Ambulant (L): ambulant late; Non ambulant (E) = non ambulant early; Non ambulant (L): non ambulant late.

**Fig 2:**
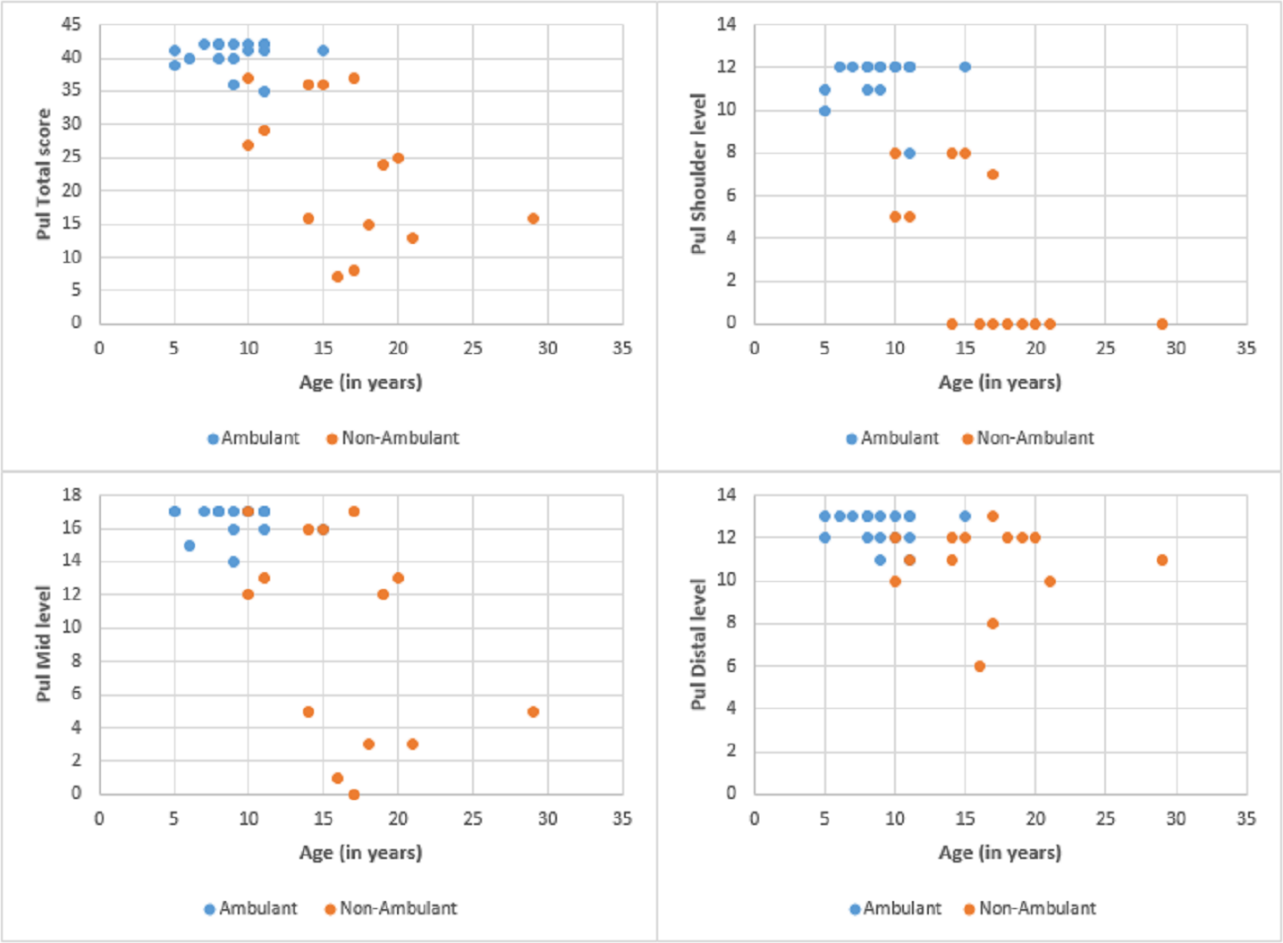
PUL and patients age. Details of the PUL scores according to age.

The total scores at baseline ranged between 6 and 42 (mean 32.7, SD: 11.3). Figs 1 and 2 report details of the PUL total scores and of the subscores for shoulder, middle and distal domains.

### Muscle MRI

None of the patients had a completely normal MRI. Fig 1 shows details of the involvement of individual muscles.

**Fig 3:**
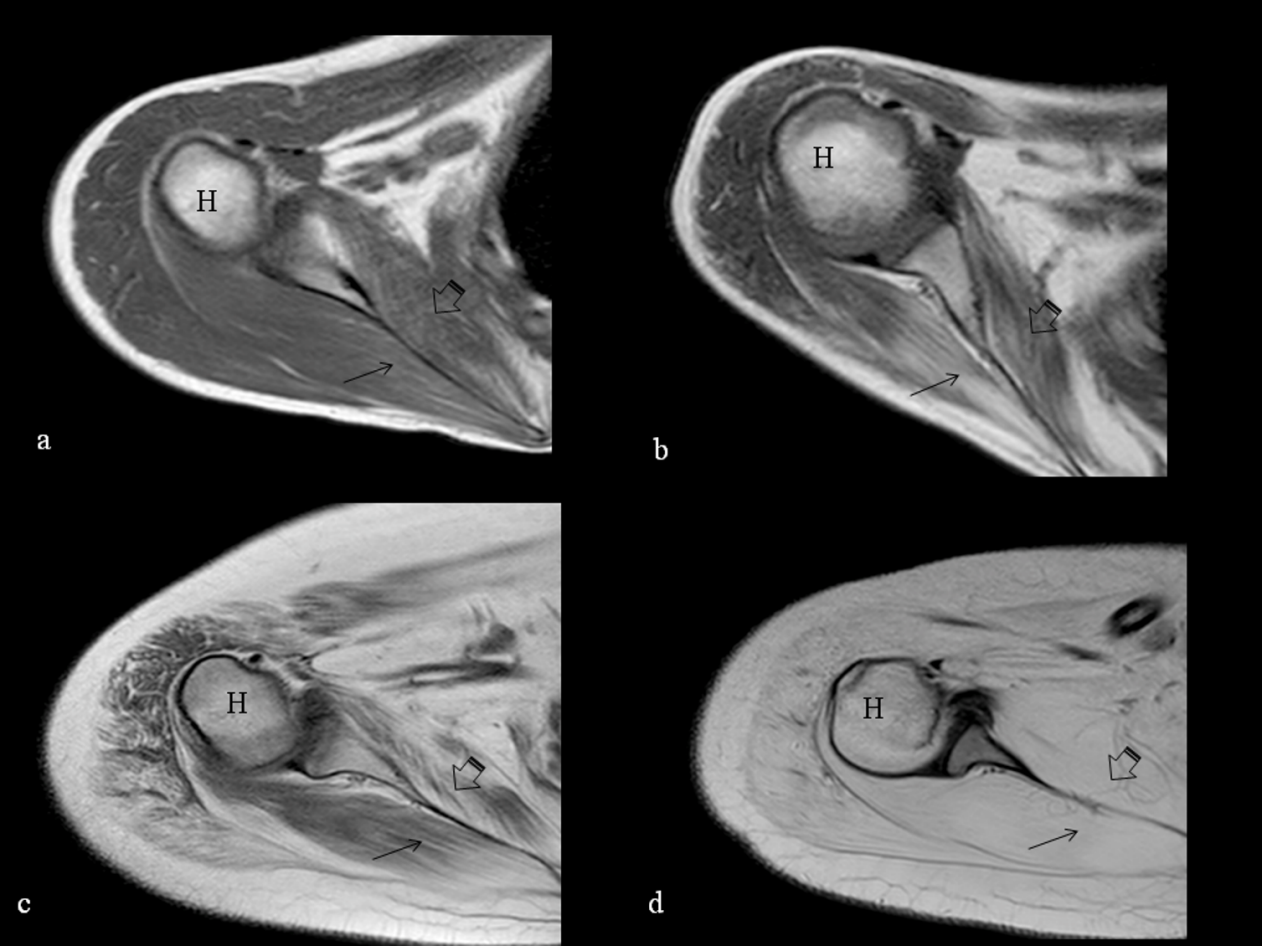
T1-weighted axial MRI of the shoulder. T1-weighted axial MRI of the shoulder at the level of the humeral head (H) showing increased signal suggestive of fatty replacement in the muscles at this level. The involvement is milder in the youngest ambulant patients aged 10 and 11 (a and b respectively) with more evident fatty replacement in the subscapularis (arrowhead) and infraspinatus (arrow) and more marked in the older non-ambulant ones, both 17 years old (c and d), with severe involvement of all the muscles shown in image d.

At shoulder level latissimus dorsi and serratus anterior, and, with one exception, the infraspinatus and the subscapularis muscles were always involved with a score of 2 or higher (100% and 96.7% respectively). Deltoid, pectoralis major and minor, and coracobrachialis were the most spared muscles. (Fig 3)

**Fig 4:**
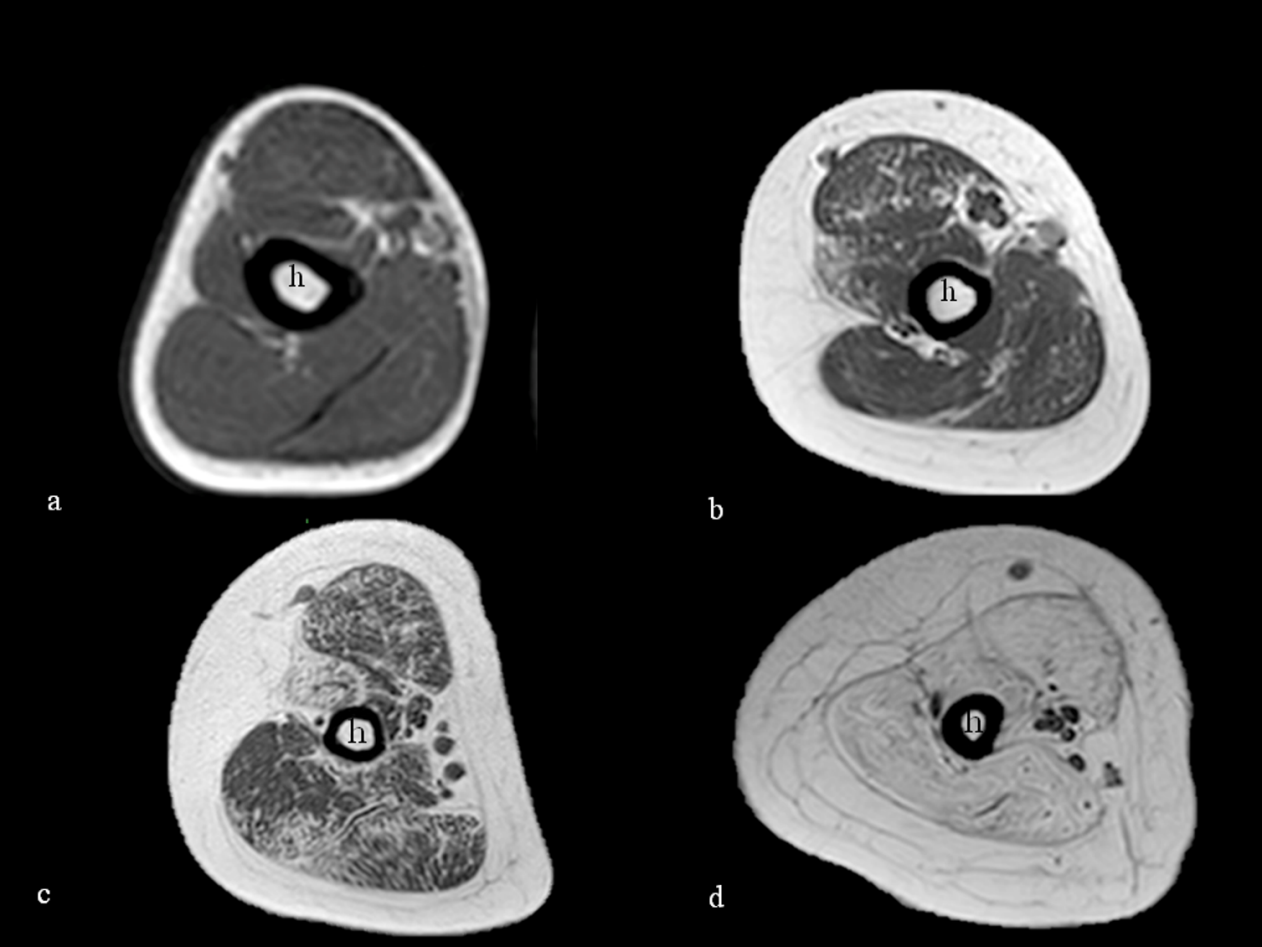
T1-weighted axial MRI of the arm. T1-weighted axial MRI of the arm at the mid-level of the humeral shaft (h) showing progressive increased signal suggestive of fatty replacement in the arm muscles. The involvement is milder in the youngest ambulant patients aged 5 and 11 (a and b respectively) and more marked in the older non-ambulant ones, both 17 years old (c and d) with severe involvement of all the muscles shown in image d.

At arm level all three major muscles often showed concordant involvement or sparing. Biceps and brachialis were affected in 18/31 (58%), and triceps in 19 (61.2%). (Fig 4)

**Fig 5:**
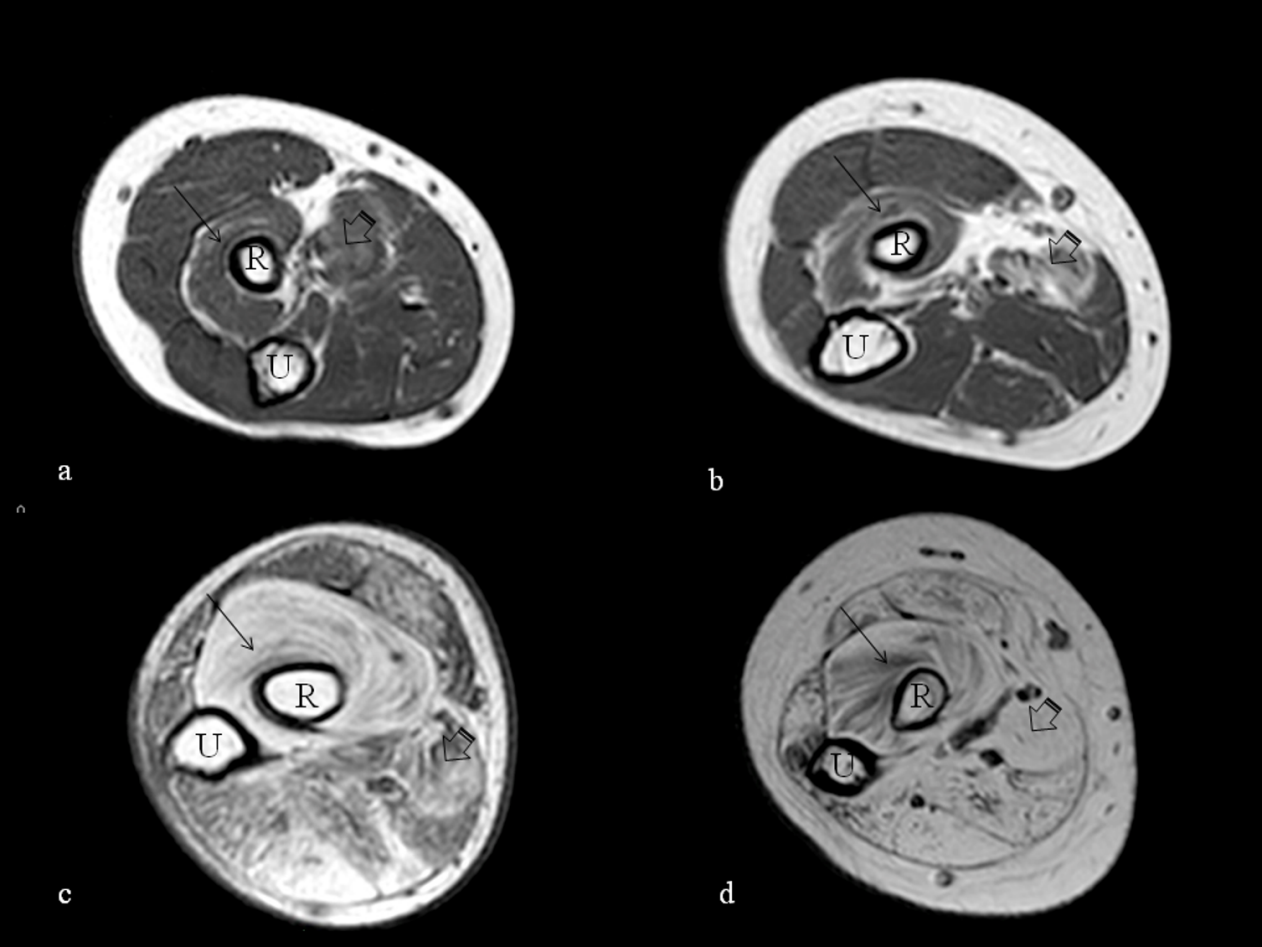
T1-weighted axial MRI of the forearm. T1-weighted axial MRI of the forearm at the level of the radial tuberosity showing increased signal suggestive of fatty replacement in the forearm muscles, especially in the supinator (arrow) and pronator teres (arrowhead). The involvement is milder in the youngest ambulant patients aged 8 and 14 (a and b respectively) and more marked in the older non-ambulant ones, both aged 17 (c and d), with severe involvement shown in image d. R (radius); U (ulna).

At forearm level the most frequently involved muscles with a score ≥2 were supinator (30/31; 96.7%, and pronator teres (27/31; 87%); brachio-radialis was also frequently involved (16/31; 51.6%). The most spared muscles were extensor carpi ulnaris, extensor pollicis longus, extensor digiti minimi, extensor digitorum, extensor carpis radialis, and the flexor pollicis longus. (Fig 5)

### PUL and MRI scores

#### Shoulder domain

Twelve patients had a full score in the shoulder domain of the PUL. On MRI all had involvement (≥2) in the latissimus dorsi, serratus anterior, infraspinatus, and subscapularis (12/12), supraspinatus and teres minor (7/12), deltoid (5/12), pectoral major (4/12), were also often involved while the other muscles were always or nearly always spared.

In the eight patients with intermediate PUL scores (8 to 11) in the shoulder domain, there was a similar MRI pattern but pectoralis minor and coraco-brachialis were also often involved (4/7).

The eleven patients with a shoulder PUL score of <8 generally showed a more diffuse and more severe involvement of all muscles.

#### Mid-level domain

Fourteen patients had a full score in the mid-level domain of the PUL. On MRI, four of the 14 had involvement (≥ 2) of all arm studied muscles (biceps, brachialis and triceps) while an additional one had only involvement of the triceps.

In the 11 patients with an intermediate mid-level score (16–12), three had all muscles spared on MRI, and the other eight had involvement (≥ 2) of all three muscles. In three cases the triceps was slightly less involved than the other two.

Six patients had very low mid-level score of the PUL, (6 or below) and on MRI all six had a diffuse and more severe involvement of all the arm muscles. In three of the six the triceps was slightly less involved.

#### Distal domain

Eleven patients had a full score of 13 in the distal domain of the PUL. On MRI, supinator (10/11) (91%) and pronator teres (9/11) (82%) were the muscles that were most frequently involved, followed by brachioradialis (4/11) (36.3%) and flexor carpi ulnaris (3/11) (27%); Extensor digiti minimi, extensor pollicis longus and flexor pollicis longus were never involved while the remaining muscles were only involved in one patient.

The remaining 20 patients had intermediate scores in the distal domain of the PUL (6 to 12). Supinator (20/20) (100%) and pronator teres (18 /20) (90%) were the most frequently affected muscles, followed by brachioradialis (13/20) (65%), flexor carpi ulnaris flexor carpi radialis, flexor digitorum superficialis and flexor digitorum profundus (10/20) (50%); palmar and flexor pollicis longus (8/20) (40%); extensior carpi ulnaris (7/20) (35%). Muscles in the extensor compartment were generally less affected (Fig 5). In 6 of the 20 there was a more diffuse involvement of all forearm muscles, that was severe in the two with lowest PUL score.

#### PUL total scores

**Fig 6:**
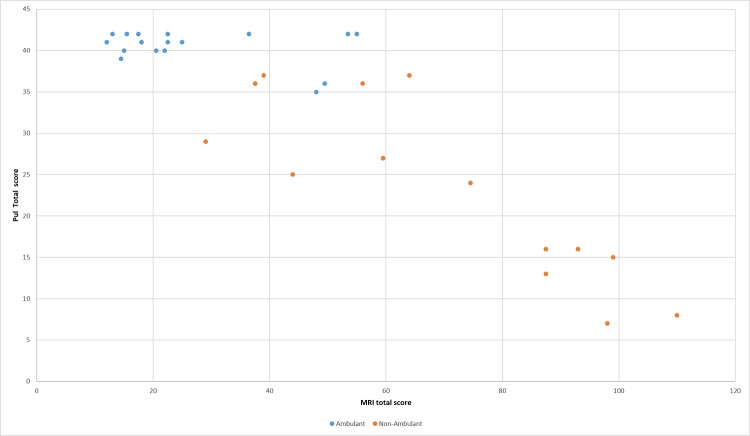
Pul and MRI total scores. Details of the PUL and MRI total scores.

**Fig 7:**
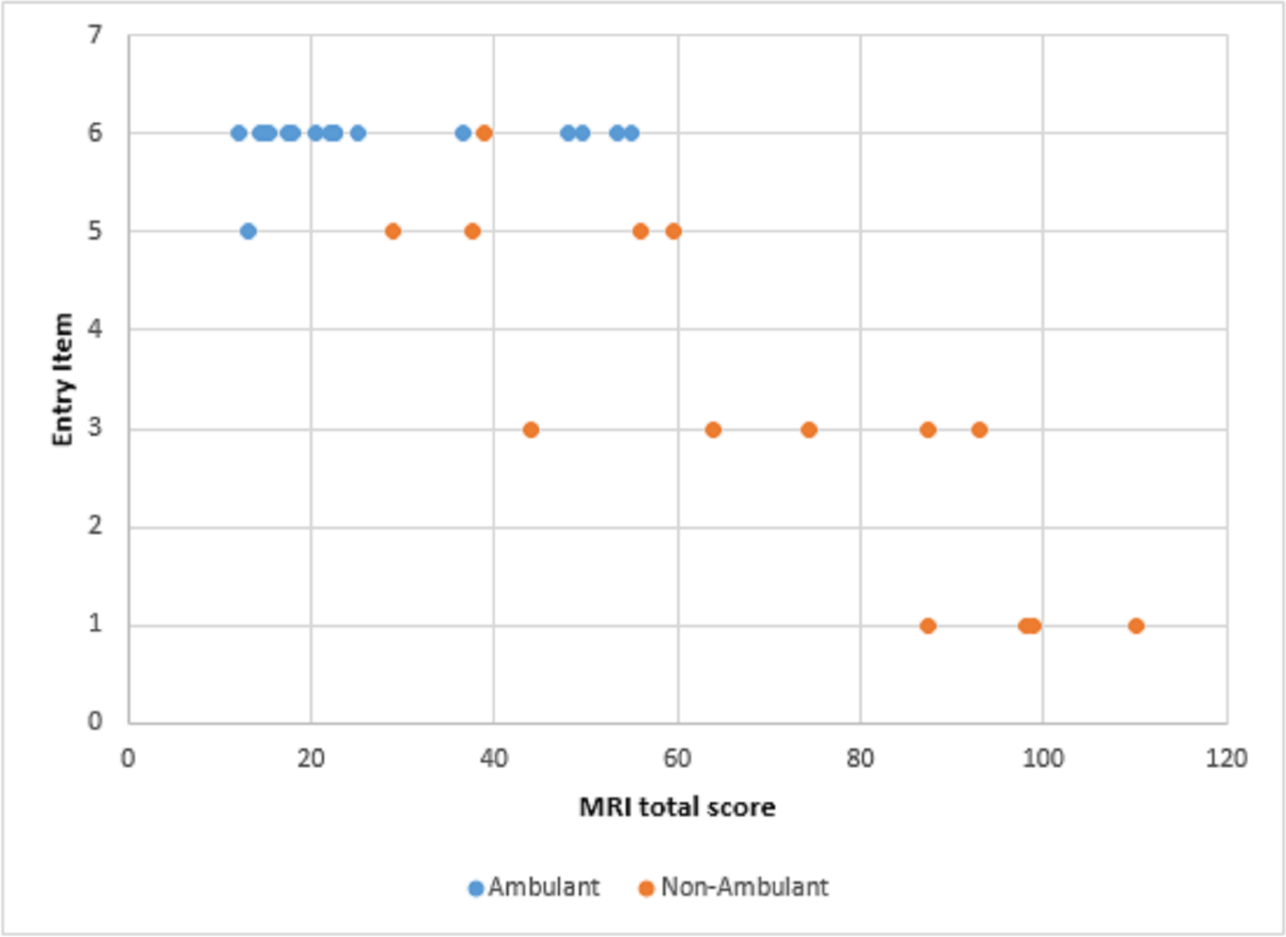
Pul entry items and MRI total scores. Details of the PUL entry items and MRI total scores. The entry items range from score 0 –no useful hand function—to score 6 full shoulder abduction–no weakness.

Figs 6 and 7 show details of the PUL total scores and entry items in relation to MRI total scores in ambulant and non ambulant patients.

## Discussion

This is the first study systematically assessing both clinical and MRI findings at shoulder, arm and forearm level in a cohort of ambulant and non-ambulant DMD patients. The use of the PUL, that provides subscores indicating the activities at shoulder, mid and distal level, allowed to identify the range of functional abilities and to establish how these related to muscle MRI findings at each level.

Some signs of muscle involvement on MRI could be detected in all the patients, even in those who had full scores on the PUL, therefore also showing full scores on the shoulder domain. More specifically, at shoulder level, some muscles in the more medial compartment, i.e. those closer to the paravertebral region, were always involved. The lack of functional impairment on the PUL in these patients probably reflects that these muscles are more involved in postural control than in the functional activities that are part of the shoulder domain of the PUL, or that other muscles are able to compensate the complex functions assessed. This is not surprising as the PUL was specifically designed to assess upper limb functional abilities rather than other functional aspects as postural control. The upper limb activities in the shoulder domain on the PUL mainly rely on other muscles (deltoid, supraspinatus, coracobrachialis and pectoralis major) that were generally spared in our patients with full PUL shoulder scores while, in these activities, the more medial muscles involved on MRI mainly play an accessory role that is probably compensated by other muscle groups.

Our findings, until now unreported to our knowledge, raise the question whether a more systematic assessment of posture and a more extensive MR protocol should be performed in these patients in order to better define early clinical and MRI signs related to the impairment of the proximal medial muscles. This should include other muscles such as trapezius, levator scapulae, and rhomboids, that are more systematically assessed in disorders with a different pattern of shoulder and paravertebral weakness, such as facioscapulohumeral muscular dystrophy [21, 22].

As expected, with decreasing PUL scores and reduced functional abilities at shoulder level, other muscles, such as deltoid, pectoral major and minor and coraco-brachialis were found to be increasingly involved on MRI reaching a stage characterized by diffuse and severe involvement of all muscles in the patients with very low scores (≤ 5) on the shoulder domain of the PUL.

At arm level, full scores in the mid-level domain of the PUL were generally associated with no or little involvement of the arm muscles on MRI. Some degree of arm muscles involvement however could already be found even in patients with small functional impairment on the mid domain PUL scores. This suggests that many of the PUL items at this level, that are clinically relevant for activities of daily living, such as feeding, etc, are grossly retained at the time the arm muscles are partly involved on MRI. These activities were lost only in the patients with the most severe and diffuse involvement of the arm muscles on MRI. It is of note that biceps, brachialis and triceps, were, with few exceptions, always concordantly involved. In the few discordant cases, triceps was generally slightly less involved than the other two.

At distal level full scores in the distal domain of the PUL were generally associated with involvement of supinator (97%) and often also of the pronator (87%). As recently reported by our group [23], these two muscles were selectively involved even in young patients with full total scores on the PUL. In these patients, including the youngest ones, the involvement on MRI was often associated with a mild restriction of the active range of movements. When we looked at the results of the distal domain on the PUL, we found that, as already reported [15], the PUL was apparently less sensitive to detect distal changes as the range of scores was relatively narrow and with one exception, were all between 10 and 13. MRI however also suggested that muscle involvement in these patients was not diffuse and rarely severe. The muscles which were more frequently involved, after supinator and pronator, were brachioradialis (16/31) (52%) and, much less frequently, the flexor compartment. It is of note that even in non-ambulant older boys and young adults the extensor muscles were, with few exceptions, often completely spared. A more diffuse and severe involvement was found only in the two patients who had scores of 8 or below. One of the limitations of this study is that we did not scan the oldest patients with severe respiratory or cardiac involvement in whom a more diffuse involvement could have been detected. Similarly, we also did not scan some of the very young children, below the age of 5, who would require sedation.

Other studies have reported detailed MRI and MRS findings in the upper limb in DMD but the results are not easily comparable to ours as they mainly focused on specific segments and, rather than focusing on pattern recognition, they aimed to quantify the level of fat replacement and muscle involvement. A more detailed analysis, using more appropriate Dixon sequences, is currently being performed in our cohort and will be reported separately. Nevertheless, although the aim of this paper was not to provide a detailed quantification of muscle involvement, it is of interest that the visual analysis used to identify patterns of involvement allowed to obtain a gross evaluation of radiological impairment that showed that the level of muscle impairment on MRI varied according to the PUL total scores and the PUL entry items.

In conclusion our results suggest that the combined assessment of more segments of the upper limbs is often useful as there is often a concomitant involvement of muscles at different levels and that patterns of muscle involvement can be detected in both ambulant and non-ambulant patients.

Early MR signs of muscle involvement could be detected even when functional abilities of the upper limbs were still normal. Despite this was a cross sectional study and we therefore cannot establish longitudinal changes, our findings showed that some muscles are more frequently involved even in younger stronger patients and that other muscles become progressively involved with increasing age and decreasing functional abilities, as recently also reported in a study using quantitative assessment of upper limbs in DMD [13]. The integrated use of functional scales and imaging also allowed to establish, at each level, the scores on the PUL that were more frequently associated with diffuse and severe involvement.

This information can be useful at the time of designing clinical trials or intervention studies to exclude or select patients or to select the most appropriate imaging protocol according to the population studied. The follow up of these patients and the results of the quantitative analysis, both in progress, are needed to provide more accurate information on longitudinal changes at all levels.

## Supporting information

S1 File PUL 2.0 original scoresheet. (DOCX)
