# Supplementary figures and images for: Functional levels and MRI patterns of muscle involvement in upper limbs in Duchenne muscular dystrophy
Source: PLoS One. 2018 Jun 20;13(6):e0199222. doi: 10.1371/journal.pone.0199222 (PMC6010282; doi:10.1371/journal.pone.0199222)

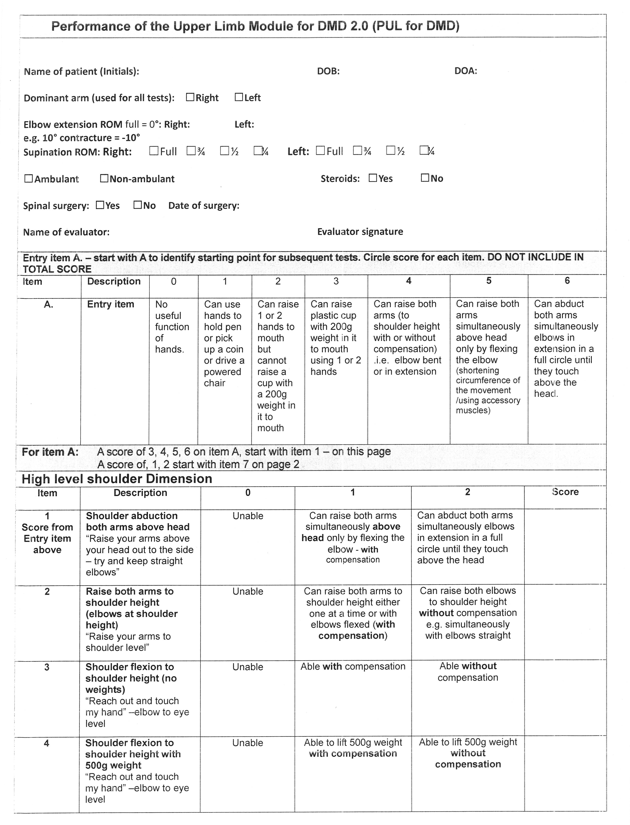


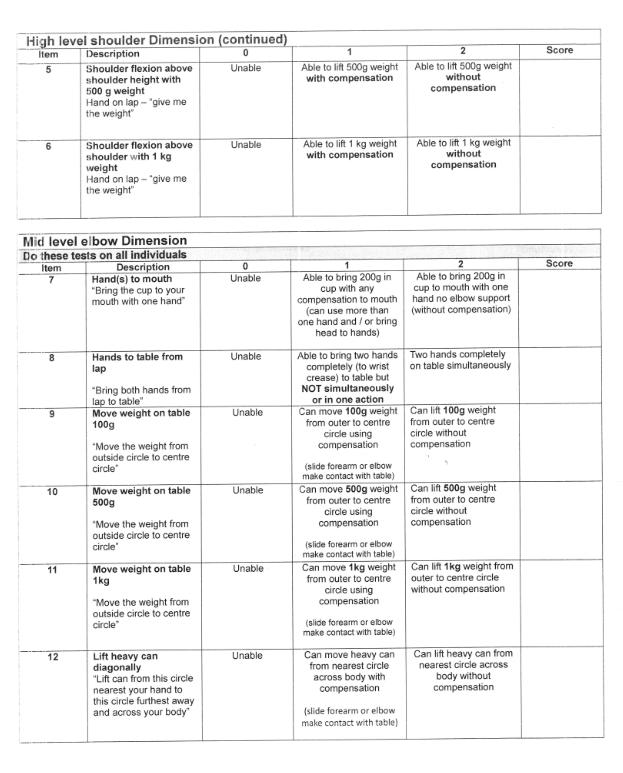


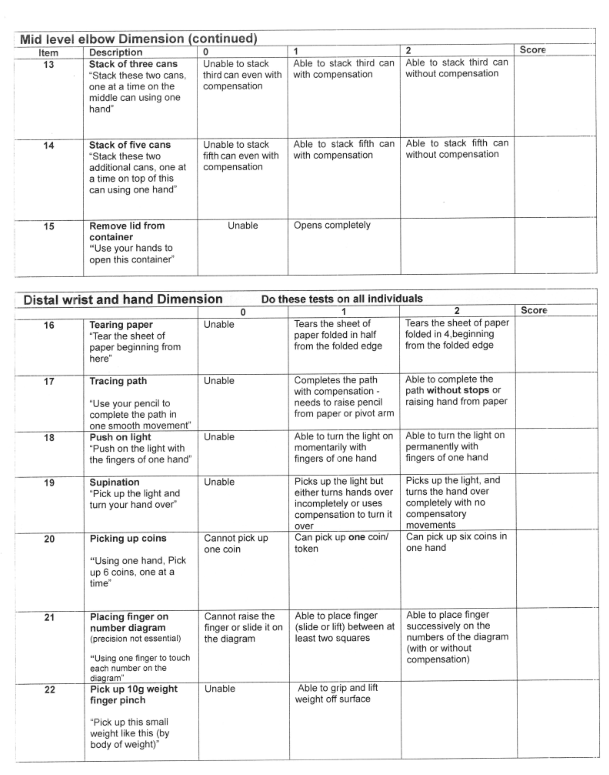


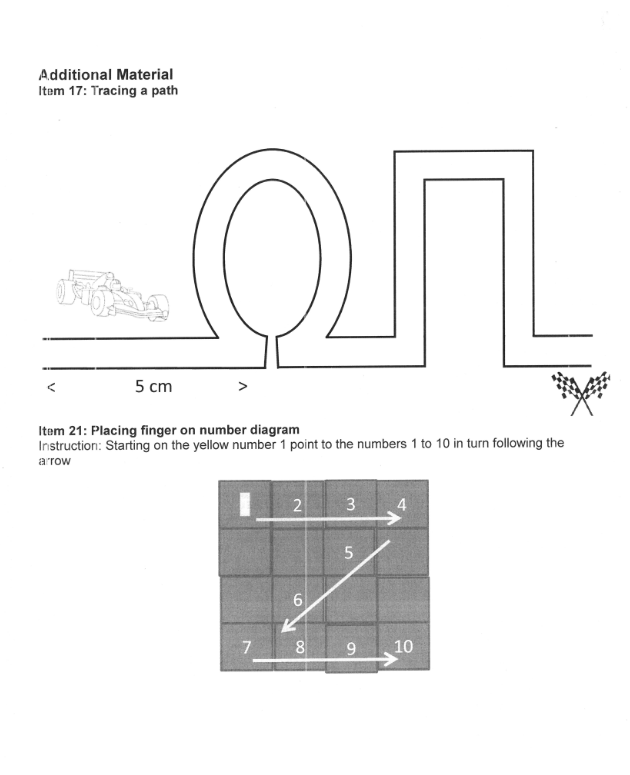

Supplement: S1 File — (DOCX) [file pone.0199222.s001.docx]
